# Xerostomia from age 50 to 90 years: prediction and prevalence in cross-sectional and longitudinal studies

**DOI:** 10.3389/froh.2025.1648038

**Published:** 2025-09-04

**Authors:** Ann-Katrin Johansson, Ridwaan Omar, Josefin Sannevik, Berit Mastrovito, Caroline Blomma, Anders Johansson

**Affiliations:** 1 Department of Clinical Dentistry – Cariology, Faculty of Medicine, University of Bergen, Bergen, Norway; 2 College of Dentistry, Kuwait University, Safat, Kuwait; 3 Department of Dentistry, Örebro County Council, Örebro, Sweden; 4 Dental Commissioning Unit, Östergötland County Council, Linköping, Sweden; 5 Department of Clinical Dentistry –Prosthodontics, Faculty of Medicine, University of Bergen, Bergen, Norway; 6 Department of Thoracic Medicine, Haukeland University Hospital, Bergen, Norway

**Keywords:** ageing, dry mouth, longitudinal, questionnaire, xerostomia

## Abstract

**Objectives:**

To describe longitudinal changes and risk factors for xerostomia in two ageing samples from age 50 to 80 and from 75 to 90.

**Material and methods:**

In 1992 and 2007, postal questionnaires were sent to the total population of 50-year-olds (born 1942, 8,888 individuals) and 75-year-olds (born 1932, 5,195 individuals), respectively. The study was repeated every 5th year up to 2022. The questions encompassed self-reports on sociodemographic domains, and perceived general and oral health. Two questions on xerostomia were included: (i) “Does your mouth feel dry during the day” and (ii) “Does your mouth feel dry at night”. The cross-sectional participation rate during the examination years ranged from 74.9% to 54.6% and was 39% in the longitudinal samples.

**Results:**

In the cross-sectional samples the response “yes, often” daytime xerostomia increased from a few percent at age 50 to 9.4% at age 80 and from 8.5% at age 75 to about 15% at age 90. The corresponding figures for reported “yes, often” nighttime xerostomia was from about 5% to 21% from age 50-to-80 and 19% to just above 24% from age 75-to-90. Figures for the longitudinal samples were similar for both conditions and cohorts. Women reported significantly higher prevalences of xerostomia than men at most of the examination points (*p* < 0.02 to *p* < 0.001). Nighttime xerostomia reported at baseline at ages 50 and 75 was the most common predictive significant risk factor for having “yes, often” both daytime and nighttime xerostomia at the end point of the study, viz. at ages 80 and 90 (OR 2.5–5.1, *p* = 0.006 to <0.001).

**Conclusions:**

“Often” xerostomia reported at baseline 30 and 15 year earlier was a common predictor for having xerostomia at ages 80 and 90 in addition to impaired general health, prescribed medication and doctor visits. Clinicians should be aware of the precipitating risk factors for xerostomia that may prevail earlier in life and therefore implement preventive strategies at an early stage.

## 1 Introduction

In a global perspective, the number of older individuals is increasing and currently forming a larger part of the world's total population than earlier. This change in diversity of the population may still be in an early phase in some countries but is ongoing or has been reached in others. In 2023, individuals ≥65 years of age represented 3.7% of the population in the least developed countries and 20% in developed ones. By 2050, these figures are expected to rise to 6.1% and 27.8%, respectively (1). As regards the very old, people aged 80 or above are estimated by 2050 to reach 434 million, representing 21% of the total population above 60 (2).

In Sweden, the number of 85-year-olds and above more than quadrupled between 1970 and 2023, from 70,000 to 300,000 and is projected to reach 750,000 individuals in 2017. In the future it is therefore likely that the cost, for example of social need, caregiving and healthcare for older individuals will increase considerably (3).

Oral disease is one of the most prevalent noncommunicable diseases affecting approximately 3.5 billion individuals worldwide (4). As the world's population steadily becomes older in combination with an increased number of teeth retained into old age, maintaining good oral healthcare faces several barriers.

In this regard, the elderly, besides more often being medically compromised also often have a high treatment need regarding oral health which frequently involves complicated and expensive restorative treatments in combination with high costs and insufficient public funding. In addition, reduced access to dental treatment due to impaired mobility and poor access to transportation, in combination with the mitigating factors of social isolation and loneliness will reduce elders' access to dental care. All of these barriers and the high perceived need for oral care may be very difficult to manage in the future (5). In relation to the foregoing, oral health is a key indicator of overall health in older age. In this regard, WHO has stated that a better integration of oral healthcare into general healthcare systems is required (6).

Xerostomia is one of the common conditions that affects many elderly persons and may result in several complications related to dental and mucosal diseases, chewing and eating problems, pain and discomfort in addition to reduced quality of life (7, 8). Prevalences of xerostomia have been reported within the range 10%–73.5% and its presence in older age depends on diverse participating factors, for example systemic diseases, medications, radiation/chemotherapy and stress (9).

The purpose of this study was to describe longitudinal changes in reported xerostomia in two ageing samples, one from age 50 to 80 and the other from 75 to 90. An additional aim was to identify risk factors, based on self-reports at baseline, for having dry mouth at the end point of the study.

## 2 Material and methods

### 2.1 Study sample

In 1992, a postal questionnaire was sent out by ordinary mail to the total population of 50-year-olds (born in 1942) living in two Swedish medium-sized counties. The study was repeated every 5th year up to 2022. The 50-year-olds in 1992 comprised 8,888 individuals out of whom 6,346 returned the questionnaire corresponding to a participation rate of 71.4%. The participation rates during the subsequent examination years, viz. 1997, 2002, 2007, 2012 and 2017, were 74.4%, 74.9%, 73.1%, 72.2% and 70.7%, respectively. In 2022, the total population of 80-year-olds was 6,299 out of which 4,355 responded giving a participation rate of 69.1% (52.1% women).

In 2007, all 75-year-olds (born in 1932) were included in the investigation. The total population of 75-year-olds in 2007 comprised 5,195 individuals out of which 3,735 responded giving a participation rate of 71.9%. The corresponding participation rates for the 2012 and 2017 examinations were 66.3% and 60.8%, respectively. In 2022, the total population of those aged 90 was 1904 and 1,040 responded which corresponds to a participation rate of 54.6% (61.0% women).

The longitudinal samples, i.e., those who responded at all the examinations, were 2,479 (54.1% women) and 758 (57.8% women) participants in the 1942 and 1932 cohorts, respectively. This corresponds to a participation rate of 39.3% for the 80-year-olds and 39.8% for the 90-year-olds.

### 2.2 Questionnaire

The questionnaire used was in the main similarly designed in all dispatches from 1992 and onwards except for some minor changes in order to be able to record altered circumstances that are normally expected to occur during ageing. The questions encompassed self-reports on sociodemographic domains, smoking habits and perceived general and oral health status. Two reminders were sent to those who did not answer.

Two questions on xerostomia were included in the questionnaire: (i) “Does your mouth feel dry during the day” and (ii) “Does your mouth feel dry at night”. The response alternatives were: (1) yes, often; (2) yes, sometimes; (3) no, seldom; (4) no, never.

### 2.3 Statistical analyses

Descriptive and analytic statistical methods were applied using IBM SPSS Statistics ver. 29. Gender differences in reported xerostomia at the different examination time points were analyzed with Mann Whitney *U*-test. Pearson chi square tests were applied for bivariate comparisons between reported xerostomia in 2022, and dichotomized tentatively associated variables that had been reported at baseline, i.e., in 1992 and 2007 for the 1942 and 1932 cohorts, respectively. Differences between daytime and nighttime xerostomia were analyzed with Wilcoxon Signed Ranks Test. Statistically significant variables according to the bivariate comparisons were entered into a multivariate logistic regression model (Forward: Conditional method) for assessment of risk factors for reporting “yes, often” xerostomia in 2022.

## 3 Results

### 3.1 Cross-sectional vs. longitudinal samples

**Figure 1:**
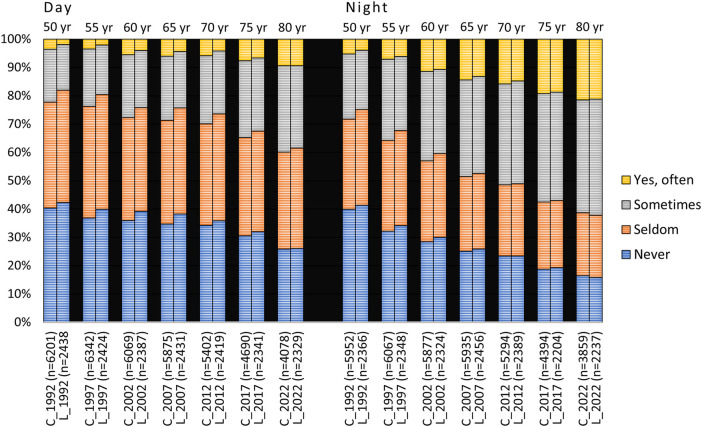
Responses to the questions “Does your mouth feel dry daytime” and “Does your mouth feel dry nighttime” from 1992 to 2022 in the 1942 cohort viz. from age 50 to 80 years. C = cross-sectional sample (*N*_total_ = 8,888, 8,764, 8,500, 8,313, 7,889, 7,204 and 6,299 for the different examination years, respectively); L = longitudinal sample (*N* = 2,479). In the figure, *n* represents the number of responders.

Responses to the question on xerostomia during the day and at night for both the cross-sectional and longitudinal samples between the ages of 50 and 80 (1942 cohort) are shown in Figure 1. The response “yes, often” daytime xerostomia increased from a few percent at age 50 to 9.4% at age 80. Cross-sectional data were quite similar to that reported in the longitudinal sample. “Yes, often” nighttime xerostomia increased from about 5% at age 50 to about 21% at age 80 and again the cross-sectional data corresponded relatively well with that reported in the longitudinal sample. If “sometimes” xerostomia was included there were increases from about 20% at age 50 to about 40% at age 80 during daytime, and at night from about 25% to about 60% at age 80. The figures were similar for both the cross-sectional and longitudinal samples (Figure 1).

**Figure 2:**
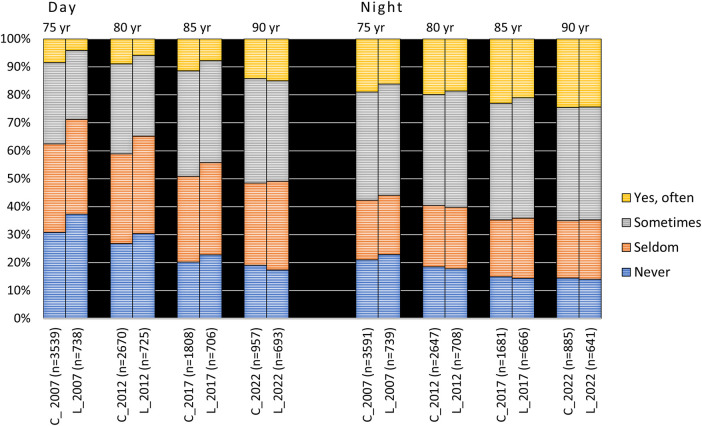
Responses to the questions “Does your mouth feel dry daytime” and “Does your mouth feel dry nighttime” from 2007 to 2022 in the 1932 cohort viz. from age 75 to 90 years. C = cross-sectional sample (*N*_total_ = 5,195; 4,404; 3,323 and 1,904 for the different examination years, respectively); L = longitudinal sample (*N* = 758). In the figure, *n* represents the number of responders.

“Yes, often” daytime xerostomia in the 75-year-olds increased from 8.5% and 4.1% in the cross-sectional and longitudinal samples, respectively, to about 15% at age 90 in both samples. Correspondingly, the figures for nighttime xerostomia were 19% and 16% in the cross-sectional and longitudinal samples, respectively, which in both cases increased to just above 24% at age 90 (Figure 2). If “sometimes” xerostomia was included, xerostomia increased from around 30% at age 75 to just over 50% at age 90 in the cross-sectional sample, and similarly for the longitudinal group.

Nighttime xerostomia was significantly higher than in daytime at all examination time points for both the 1942 and 1932 cohorts and genders (*p* < 0.001).

### 3.2 Gender differences

**Figure 3:**
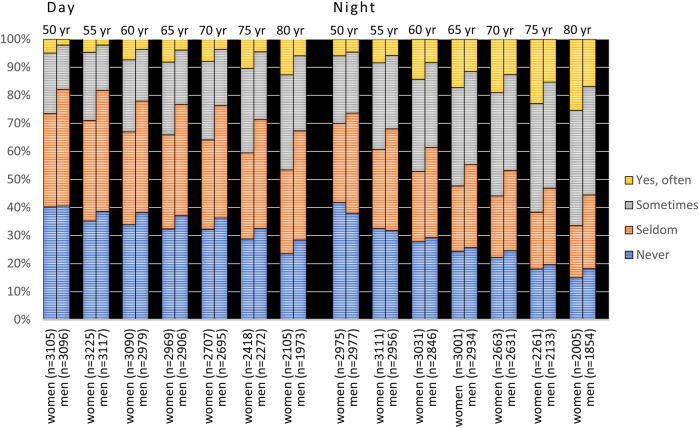
Cross-sectional: responses to the questions “Does your mouth feel dry daytime” and “Does your mouth feel dry nighttime” from 1992 to 2022 in the 1942 cohort viz. from age 50 to 80 years stratified by gender. In the figure, *n* represents the number of responders.

**Figure 4:**
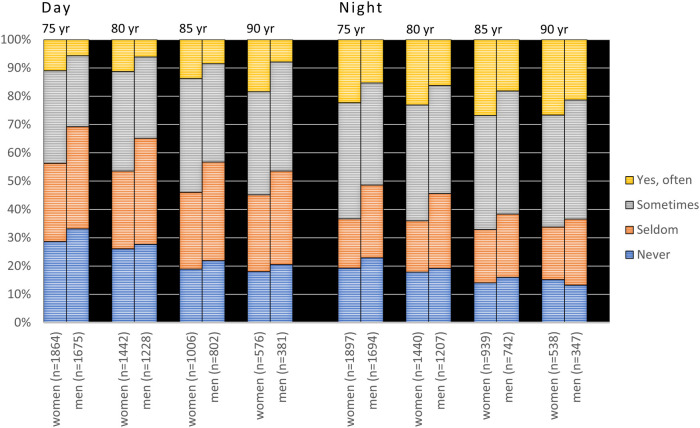
Cross-sectional sample: responses to the questions “Does your mouth feel dry daytime” and “Does your mouth feel dry nighttime” from 2007 to 2022 in the 1932 cohort viz. from age 75 to 90 years stratified by gender. In the figure, *n* represents the number of responders.

**Figure 5:**
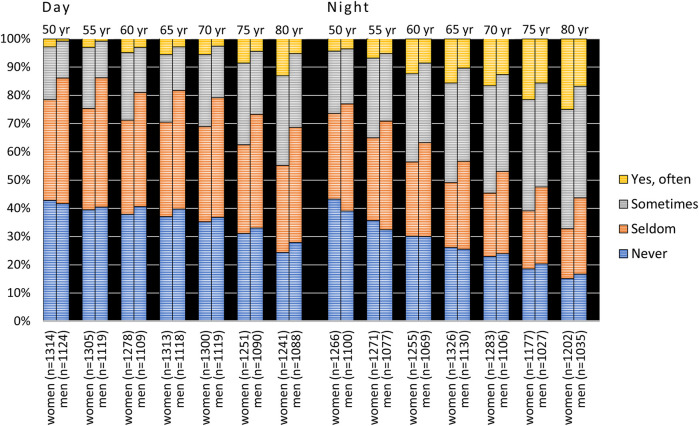
Longitudinal sample: responses to the questions “Does your mouth feel dry daytime” and “Does your mouth feel dry nighttime” from 1992 to 2022 in the 1942 cohort viz. from age 50 to 80 years stratified by gender (*N*_total_ = 2,479). In the figure, *n* represents the number of responders.

**Figure 6:**
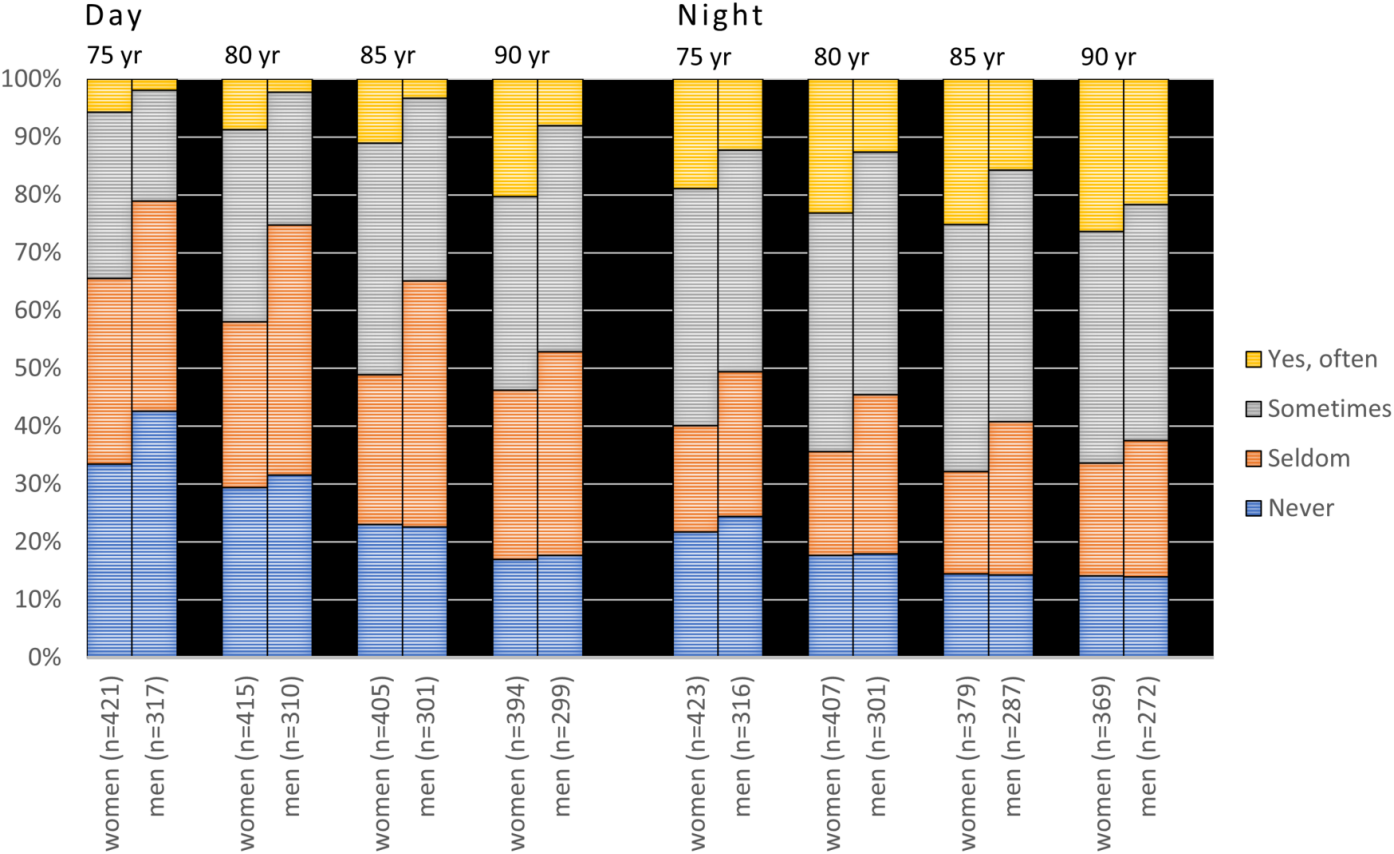
Longitudinal sample: responses to the questions “Does your mouth feel dry daytime” and “Does your mouth feel dry nighttime” from 2007 to 2022 in the 1932 cohort viz. from age 75 to 90 years stratified by gender (*N*_total_ = 758). In the figure, *n* represents the number of responders.

Women reported significantly higher prevalences of xerostomia than men at all examination points in cross-sectional samples for both cohorts (*p* < 0.001, Figures 3, 4) except for nighttime in the 90-year-olds in 2022 (Figure 4) (Mann Whitney *U*-test). In the longitudinal sample and for the 1942 cohort, women reported significantly more xerostomia at all time points (*p* < 0.02 to *p* < 0.001) except for at age 50 (for both daytime and nighttime) and nighttime at age 55 (Figure 5). For the 1932 cohort longitudinal sample, women reported significantly more xerostomia at all examination points than men (*p* < 0.01 to *p* < 0.001) except for at night at age 90 (Figure 6) (Mann Whitney *U*-test).

### 3.3 Predictors for xerostomia

In the bivariate analysis, a large number of significant differences were found between “yes, often” xerostomia reported in 2022, and tentatively associated variables reported in 1992 for the 1942 cohort (Table 1) and in 2007 for the 1932 cohort (Table 2). Those variables with *p*-value of <0.05 were entered into an adjusted regression analysis (Tables 3, 4).

**Table 1:** Analysis between reported xerostomia (1 = yes, often vs. 2 = yes, sometimes, no, seldom/never) in 2022 and variables reported in the 50-year-old longitudinal sample in 1992 (now 80-year-olds; 1942 cohort; *n* = 2,479). (Pearson chi square).

Variables	Dichotomization	Daytime	Nighttime
		*p*	*p*
Socio-demographic:			
Gender	1 = man; 2 = woman	<0.001	<0.001
Birthplace	1 = Sweden; 2 = other country	NS	NS
Residency	1 = large or small city; 2 = countryside	NS	NS
Education	1 = elementary school (6 years); 2 = higher secondary school, gymnasium, university	NS	0.03
Marital status	1 = married/cohabiting; 2 = unmarried/divorced/widow(er)	0.03	NS
General health:			
Perceived full health	1 = yes, absolutely/by and large; 2 = no, not especially/absolutely not	<0.001	<0.001
Own health compared to peers	1 = yes, much better/better/equally good; 2 = no, worse/much worse	<0.001	0.007
Medication (prescribed) last 14 days	1 = yes; 2 = no	<0.001	<0.001
Doctor visit last 3 months	1 = yes, several/some/one times; 2 = no	<0.001	<0.001
Smoking	1 = smoking daily/occasionally; stopped; 2 = never smoked	NS	NS
Oral health:			
Teeth satisfaction	1 = yes, very satisfied/by and large satisfied; 2 = no, not especially satisfied/absolutely not satisfied	<0.001	<0.001
Ability to chew all kind of food	1 = very good; 2 = rather good/not so good/bad	<0.001	<0.001
Satisfaction with teeth esthetics	1 = yes, very pleased/by and large pleased; 2 = not especially/absolutely not	<0.001	<0.001
Number of own teeth	1 = all teeth left/missing a single tooth; 2 = missing rather many teeth/almost no teeth left/edentulous	<0.001	<0.001
Xerostomia daytime	1 = yes, often; 2 = yes, sometimes, no, seldom/never	<0.001	<0.001
Xerostomia nighttime	1 = yes, often; 2 = yes, sometimes, no, seldom/never	<0.001	<0.001
Do you have trouble with:			
Burning mouth	1 = no trouble: 2 = some/rather much/great troubles	<0.001	<0.001
Oral wounds or blisters	1 = no trouble: 2 = some/rather much/great troubles	0.008	<0.001
Taste changes	1 = no trouble: 2 = some/rather much/great troubles	<0.001	<0.001
TMJ pain	1 = no trouble: 2 = some/rather much/great troubles	<0.001	<0.001
TMJ sound	1 = no trouble: 2 = some/rather much/great troubles	<0.001	0.006
Mouth opening	1 = no trouble: 2 = some/rather much/great troubles	<0.001	NS
Bruxism	1 = no trouble: 2 = some/rather much/great troubles	0.001	0.01
Bleeding gums	1 = no trouble: 2 = some/rather much/great troubles	0.002	<0.001
Bad breath	1 = no trouble: 2 = some/rather much/great troubles	<0.001	<0.001
Tooth sensitivity	1 = no trouble: 2 = some/rather much/great troubles	<0.001	<0.001

**Table 2:** Analysis between reported xerostomia (1 = yes, often vs. 2 = yes, sometimes, no, seldom/never) in 2022 and variables reported in the 75-year-old longitudinal sample in 2007 (now 90-year-olds; 1932 cohort; *n* = 758). (Pearson chi square).

Variables	Dichotomization	Daytime	Nighttime
		*p*	*p*
Socio-demographic:			
Gender	1 = man; 2 = woman	<0.001	NS
Birthplace	1 = Sweden; 2 = other country	NS	NS
Residency	1 = large or small city; 2 = countryside	NS	NS
Education	1 = elementary school (6 years); 2 = higher secondary school, gymnasium, university	NS	NS
Marital status	1 = married/cohabiting; 2 = unmarried/divorced/widow(er)	NS	NS
General health:			
Perceived full health	1 = yes, absolutely/by and large; 2 = no, not especially/absolutely not;	<0.001	0.004
Own health compared to peers	1 = yes, much better/better/equally good; 2 = no, worse/much worse	<0.001	NS
Medication (prescribed) last 14 days	1 = yes; 2 = no	0.009	0.03
Doctor visit last 3 months	1 = yes, several/some/one times; 2 = no	0.01	NS
Smoking	1 = smoking daily/occasionally; stopped; 2 = never smoked	NS	NS
Oral health:			
Teeth satisfaction	1 = yes, very satisfied/by and large satisfied; 2 = no, not especially satisfied/absolutely not satisfied;	<0.001	0.03
Ability to chew all kind of food	1 = very good; 2 = rather good/not so good/bad	0.03	NS
Satisfaction with teeth esthetics	1 = yes, very pleased/by and large pleased; 2 = not especially/absolutely not;	NS	NS
Number of own teeth	1 = all teeth left/missing a single tooth; 2 = missing rather many teeth/almost no teeth left/edentulous	0.03	NS
Xerostomia daytime	1 = yes, often; 2 = yes, sometimes, no, seldom/never	<0.001	<0.001
Xerostomia nighttime	1 = yes, often; 2 = yes, sometimes, no, seldom/never	<0.001	<0.001
Do you have trouble with:			
Burning mouth	1 = no trouble: 2 = some/rather much/great troubles	<0.001	<0.001
Oral wounds or blisters	1 = no trouble: 2 = some/rather much/great troubles	<0.001	0.01
Taste changes	1 = no trouble: 2 = some/rather much/great troubles	0.005	0.10
TMJ pain	1 = no trouble: 2 = some/rather much/great troubles	0.03	NS
TMJ sound	1 = no trouble: 2 = some/rather much/great troubles	0.01	NS
Mouth opening	1 = no trouble: 2 = some/rather much/great troubles	0.007	NS
Bruxism	1 = no trouble: 2 = some/rather much/great troubles	0.004	<0.05
Bleeding gums	1 = no trouble: 2 = some/rather much/great troubles	0.02	NS
Bad breath	1 = no trouble: 2 = some/rather much/great troubles	NS	NS
Tooth sensitivity	1 = no trouble: 2 = some/rather much/great troubles	0.02	0.01

**Table 3:** Adjusted regression analysis (forward: conditional method) between reported daytime and nighttime xerostomia (1 = yes, often vs. 2 = yes, sometimes, no, seldom/never) in 2022 and variables reported in the 50-year-old longitudinal sample in 1992 (now 80-year-olds; 1942 cohort; *n* = 2,479).

Variables	Daytime			Nighttime		
	Adjusted^a^			Adjusted^b^		
	OR	*p*	95% CI	OR	*p*	95% CI
Socio-demographic:						
Gender (ref. woman)	2.4	<0.001	1.7, 3.3	1.6	<0.001	1.3, 2.0
Education (ref. elementary school)	X		–	1.4	0.005	1.1, 1.7
Marital status (ref. unmarried/divorced/widow)	NS	–	–	X	–	–
General health:						
Perceived full health (ref. no)	2.5	<0.001	1.6, 3.9	NS	–	–
Own health compared to peers (ref. worse)	NS	–	–	NS	–	–
Medication (prescribed) last 14 days (ref. yes)	1.5	0.009	1.1, 2.1	NS	–	–
Doctor visit last 3 months (ref. yes)	NS	–	–	1.4	0.002	1.1, 1.8
Oral health:						
Teeth satisfaction (ref. not satisfied)	NS	–	–	NS	–	–
Ability to chew all kind of food (ref. not good)	NS	–	–	NS	–	–
Satisfaction with teeth esthetics (ref. not satisfied)	NS	–	–	NS	–	–
Number of own teeth (ref. many missing/edentulous)	1.7	0.004	1.2, 2.4	NS	–	–
Xerostomia daytime (ref. yes, often)	NS	–	–	NS	–	–
Xerostomia nighttime (ref. yes, often)	4.1	<0.001	2.5, 6.8	5.1	<0.001	3.3, 8.0
Do you have trouble with:						
Burning mouth (ref. yes, have trouble)	NS	–	–	NS	–	–
Oral wounds or blisters (ref. yes, have trouble)	NS	–	–	1.4	0.02	1.1, 1.9
Taste changes (ref. yes, have trouble)	2.0	0.005	1.2, 3.2	NS	–	–
TMJ pain (ref. yes, have trouble)	1.7	0.02	1.1, 2.6	1.5	0.02	1.1, 2.1
TMJ sound (ref. yes, have trouble)	NS	–	–	NS	–	–
Mouth opening (ref. yes, have trouble)	NS	–	–	X	–	–
Bruxism (ref. yes, have trouble)	NS	–	–	NS	–	–
Bleeding gums (ref. yes, have trouble)	NS	–	–	1.3	0.01	1.1, 1.6
Bad breath (ref. yes, have trouble)	NS	–	–	NS	–	–
Tooth sensitivity (ref. yes, have trouble)	NS	–	–	1.3	0.02	1.1, 1.6

NS, not significant; X, not included in the adjusted analysis. Number of individuals with positive response to “yes, often”: Daytime *n* = 219; Nighttime *n* = 475.

a Nagelkerke R Square 0.14.

b Nagelkerke R Square 0.09.

**Table 4:** Adjusted regression analysis (forward: conditional method) between reported daytime and nighttime xerostomia (1 = yes, often vs. 2 = yes, sometimes, no seldom/never) in 2022 and variables reported in the 75-year-old longitudinal sample in 2007 (now 90-year-olds; 1932 cohort; *n* = 758).

Variables	Daytime			Nighttime		
	Adjusted^a^			Adjusted^b^		
	OR	*p*	95% CI	OR	*p*	95% CI
Socio-demographic:						
Gender (ref. woman)	2.5	0.002	1.4, 4.5	X	–	–
General health:						
Perceived full health (ref. no)	2.8	<0.001	1.6,4.9	NS	–	–
Own health compared to peers (ref. worse)	NS	–	–	–	–	–
Medication (prescribed) last 14 days (ref. yes)	NS	–	–	1.7	0.03	1.0, 2.6
Doctor visit last 3 months (ref. yes)	NS	–	–	X	–	–
Oral health:						
Teeth satisfaction (ref. not satisfied)	NS	–	–	NS	–	–
Ability to chew all kind of food (ref. not good)	NS	–	–	X	–	–
Number of own teeth (ref. many missing/edentulous)	1.8	0.03	1.1, 3.0	X	–	–
Xerostomia daytime (ref. yes, often)	2.6	<0.05	1.0, 6.6	2.9	0.03	1.1, 7.3
Xerostomia nighttime (ref. yes, often)	2.5	0.006	1.3, 4.6	4.5	<0.001	2.8, 7.4
Do you have trouble with:						
Burning mouth (ref. yes, have trouble)	NS	–	–	3.3	0.002	1.5, 7.4
Oral wounds or blisters (ref. yes, have trouble)	NS	–	–	NS	–	–
Taste changes (ref. yes, have trouble)	NS	–	–	NS	–	–
TMJ pain (ref. yes, have trouble)	NS	–	–	X	–	–
TMJ sound (ref. yes, have trouble)	NS	–	–	X	–	–
Mouth opening (ref. yes, have trouble)	NS	–	–	X	–	–
Bruxism (ref. yes, have trouble)	NS	–	–	NS	–	–
Bleeding gums (ref. yes, have trouble)	1.7	<0.05	1.0, 2.9	X	–	–
Tooth sensitivity (ref. yes, have trouble)	NS	–	–	NS	–	–

NS, not significant; X, not included in the adjusted analysis.

a Nagelkerke R Square 0.21.

b Nagelkerke R Square 0.20.

For the 80-year-olds (1942 cohort) the strongest predictor for reporting “yes, often” xerostomia at daytime in 2022 was the presence of nighttime xerostomia in 1992 (OR 4.1, CI 2.5–6.8) followed, in order of decreasing odds ratios, by impaired general health (OR 2.5, CI 1.6–3.9), female gender (OR 2.4, CI 1.7–3.3), taste changes (OR 2.0, CI 1.2–3.2), smaller number of own teeth (OR 1.7, CI 1.2–2.4), TMJ pain (OR 1.7, CI 1.1–2.6) and use of prescribed medication (OR 1.5, CI 1.1–2.1). Nighttime “often xerostomia” in 2022 was predicted by having nighttime xerostomia in 1992 (OR 5.1, CI 3.3–8.0), female gender (OR 1.6, CI 1.3–2.0), TMJ pain (OR 1.5, CI 1.1–2.1), doctors visit in last 3 months (OR 1.4, CI 1.1–1.8), oral wounds or blisters (OR 1.4, CI 1.1–1.9), bleeding gums (OR 1.3, CI 1.1–1.6) and tooth sensitivity (OR 1.3, CI 1.1–1.6) (Table 3).

The corresponding figures for the 90-year-olds (1932 cohort) for having “yes, often” xerostomia daytime in 2022 was reported impaired general health in 2007 (OR 2.8, CI 1.6–4.9), daytime xerostomia (OR 2.6, CI 1.0–6.6), nighttime xerostomia (OR 2.5, CI 1.3–4.6), female gender (OR 2.5, CI 1.4–4.5), smaller number of own teeth (OR 1.8, CI 1.1–3.0) and bleeding gums (OR 1.7, CI 1.0–2.9). Nighttime “yes, often” xerostomia in 2022 was predicted by having had nighttime xerostomia in 2007 (OR 4.5, CI 2.8–7.4), burning mouth (OR 3.3, CI 1.5–7.4), xerostomia daytime (OR 2.9, CI 1.1–7.3) and prescribed medication (OR 1.7) (Table 4).

## 4 Discussion

This study shows that xerostomia increases steadily by age and is more prevalent at night and among women. The strongest and most commonly-reported predictive factor for having “often” xerostomia at ages 80 and 90 based on reports from 30 to 15 year earlier, at ages 50 and 75, respectively, were having nighttime xerostomia at baseline, in addition to female gender, impaired general health and prescribed medication/doctor visits. Several oral health related variables were also predictive for xerostomia at both 80 and 90 years of age.

### 4.1 Participation rate

Considering the old age of the participants, their participation rate must be considered good considering that close to 70% of the 80-year-olds responded while 55% of the 90-year-olds did. In the total population of 80-year-olds in Sweden in 2022, women constituted 52.8% and 90-year-olds constituted 64.2% (10). This corresponds quite well with the participation frequencies both in the cross-sectional samples (80 years 52.1%; 90 years 61.0%) and in the longitudinal samples (80 years 54.1%; 90 years 57.8%) of our study. Thus, and based on gender, our selected samples reflected quite well the composition of the total population in Sweden with the exception of a small underrepresentation of women among the 90-year-olds.

### 4.2 Prevalence of xerostomia

That xerostomia increases with age is a well-known fact (11) but reports of dry mouth in the very old are scarce. A literature review of xerostomia that included 12 papers on the elderly aged 60–96 years, prevalence rates of xerostomia ranged from 10% to 73.5% (12). This wide range was due to the examined samples being not exclusively population-based but selected samples having diverse predicating factors causing xerostomia. In a study of 270 Finnish home care people aged ≥75 year (mean 84.5), 56% answered affirmatively to the question whether they had “occasional xerostomia” (44%) or “continuous xerostomia” (12%) (13). These figures are analogous with the findings in our study if “sometimes” and “often” xerostomia are included, viz. 60% at age 80% and 50% at age 90. In another study of 1,286 Japanese women aged 75–84 years (mean 78.4), 38.8% reported xerostomia in response to the question “does your mouth feel dry?” (14). This figure would approach our results if “sometimes” together with “often” xerostomia are included.

In contrast to the overall majority of reports on xerostomia, we divided the question on xerostomia according to daytime and nighttime experiences. Nighttime xerostomia was consistently statistically higher than during daytime. This is a well-known fact, but its biological basis and significance are unclear although it could at least partly be explained be circadian variations in salivary flow or polypharmacy during evening hours (15). Nevertheless, it is important to record mouth dryness experienced both during daytime and nighttime during sleep as elaborated below.

### 4.3 Predictors/risk factors for xerostomia

This study showed that it was possible to predict the presence of “often” xerostomia based on reports from decades earlier. The most consistent risk factor found for having xerostomia at the end point of the study in 2022 was reported nighttime xerostomia at baseline, viz. 30 and 15 years back for the 1942 and 1932 cohorts, respectively. Female gender, impaired general health, more frequent doctor visits/intake of prescribed medicines, in addition to a number of reported intraoral problems were other risk factors for having mouth dryness. These associations have previously been well-documented, albeit in mainly cross-sectional studies (7–9, 16, 17), but as these conditions were predictive for reported frequent xerostomia decades later, it opens up the justification that preventive measures could be implemented at an early stage. It should be noted, however, that some of the conditions, like for example impaired general health, could be both a cause and a consequence of xerostomia.

The prevalence of xerostomia, as reported in this study, is maybe even higher among the non-responders in this study. There are several reasons for such a potential explanation but may include higher frequencies of persons with impaired general health/intraoral problems and prescribed medications among the non-responders, all of which increase the risk for xerostomia. Therefore, it is possible that the total prevalence of xerostomia among the elderly in reality is even higher than reported in this study.

Furthermore, neuropathic origins of xerostomia, where patients frequently report subjective dry mouth despite normal salivary flow, are increasingly recognized in the literature and termed as “neuropathic xerostomia”. This is especially relevant in relation to conditions such as Burning Mouth Syndrome (18).

### 4.4 Preventive strategies

It is recommended that all patients, especially those over the age of 50 or at risk of dry mouth, are screened for the presence of xerostomia during routine clinical examinations by using simple questions. It seems that our questions used in this study i.e., “Does your mouth feel dry during the day” and “Does your mouth feel dry at night”, are appropriate to use and serves the purpose of detecting xerostomia. If xerostomia is reported, an objective measurement of salivary flow should be considered and if hyposalivation is present, adequate preventive strategies should be implemented.

A comprehensive medical history is always recommended and may result in a referral to collaborative care and possible review/changes of the patient's medication.

In addition, the patient should be given information by the dental profession regarding dry mouth and its possible long-term effect on oral health and function. Relevant preventive measures aimed at easing the effect of the condition may involve advice regarding oral hygiene care as well as dietary and fluoride recommendations and, if needed, also recommendations about the use of saliva stimulants/substitutes or other ways of easing the discomfort of dry mouth.

### 4.5 Limitations of the study

One key limitation of this study is the lack of objective measurements of salivary flow rates. Although xerostomia is often associated with hyposalivation, the two do not always correspond. Relying solely on self-reported symptoms without objective confirmation of salivary flow may therefore have led to inaccurate assumptions regarding the physio-pathological effect of dry mouth on general and oral health. Additionally, the xerostomia assessment questions used in this study were originally developed in 1992 and have not been formally validated or tested for their psychometric properties. This raises concerns about the reliability and accuracy of the self-reported data. Another limitation is the absence of detailed information on potential confounding factors such as antidepressant use, diabetes, and polypharmacy—conditions and treatments that are prevalent among older adults and known to influence salivary function. The questionnaire included only a general question about the use of prescribed medications, without specifying medication types or dosages, limiting our ability to control for these important variables. There is also a risk of selection bias, as the participants who completed the questionnaire were likely healthier and more motivated than non-respondents. Those who did not respond may have included a higher proportion of individuals with medical or cognitive impairments—groups in which xerostomia may be more common. As a result, the prevalence of xerostomia reported in this study may underestimate its true rate in the broader elderly population. Finally, the logistic regression analyses may be affected by overfitting, particularly given the number of variables considered relative to the sample size. This could lead to inflated estimates of associations and limits the generalizability of the predictive results. Therefore, these findings should be interpreted with caution.

## 5 Conclusions

In conclusion, xerostomia is relatively uncommon at age 50 but increases steadily during ageing. Clinicians should be aware of the precipitating risk factors that prevail earlier in life and therefore implement management strategies in order to prevent the continuing suffering related to quality of life and oral diseases that xerostomia may cause.

## Data Availability

The datasets presented in this article are not readily available because the data that support the findings of this study are available upon reasonable request. Requests to access the datasets should be directed to josefin.sannevik@regionorebrolan.se.

## References

[1] United Nations Department of Economic and Social Affairs, Population Division. World Population Ageing 2023: Challenges and Opportunities of Population Ageing in the Least Developed Countries. New York: DESA Publications (2023).

[2] UN Department of Economic and Social Affairs Social Inclusion. Population Ageing and Sustainable Development. New York: Division for Inclusive Social Development (DISD) (2017).

[3] Sweden Statistics. Sveriges Framtida Befolkning 2024–2070. Available online at: https://www.scb.se/contentassets/548afb898d0a46419ec6f01189811cc2/be0401_2024i70_br_be51br2401.pdf (Accessed March 5, 2025).

[4] WHO. Global Strategy and Action Plan on Oral Health 2023–2030. Geneva: World Health Organization (2024). Available online at: 9789240090538-eng.pdf (Accessed March 5, 2025).

[5] Peres MA Macpherson LMD Weyant RJ Daly B Venturelli R Mathur MR Oral diseases: a global public health challenge. Lancet. (2019) 394(10194):249–60. 10.1016/S0140-6736(19)31146-8. Erratum in: Lancet. (2019) 394(10203):1010. doi: 10.1016/S0140−6736(19)32079−3 31327369

[6] UN Decade of Healthy Ageing: Plan of Action 2021–2030. Available online at: decade-proposal-final-apr2020-en.pdf (Accessed March 5, 2025).

[7] Johansson AK Omar R Mastrovito B Sannevik J Carlsson GE Johansson A. Prediction of xerostomia in a 75-year-old population: a 25-year longitudinal study. J Dent. (2022) 118:104056. 10.1016/j.jdent.2022.104056 35121136

[8] Locker D. Dental status, xerostomia and the oral health-related quality of life of an elderly institutionalized population. Spec Care Dentist. (2003) 23:86–93. 10.1111/j.1754-4505.2003.tb01667.x 14650556

[9] Sutarjo FN Rinthani MF Brahmanikanya GL Parmadiati AE Radithia D Mahdani FY. Common precipitating factors of xerostomia in elderly. JHASNU. (2024) 14:011–6. 10.1055/s-0043-1762916

[10] Sweden statistics. Population by Region, Marital Status, Age And Sex. Year 1968–2024. Solna; Örebro: PxWeb (2025).

[11] Agostini BA Cericato GO Silveira ERD Nascimento GG Costa FDS Thomson WM How common is dry mouth? Systematic review and meta-regression analysis of prevalence estimates. Braz Dent J. (2018) 29:606–18. 10.1590/0103-6440201802302 30517485

[12] Kamińska-Pikiewicz K Bachanek T Chałas R. The incidence of oral dryness in people over 65 years living in Lublin. Curr Issues Pharm Med Sci. (2015) 28:250–3. 10.1515/cipms-2015-0082

[13] Viljakainen S Nykänen I Ahonen R Komulainen K Suominen AL Hartikainen S Xerostomia among older home care clients. Community Dent Oral Epidemiol. (2016) 44:232–8. 10.1111/cdoe.12210 26739925

[14] Ohara Y Hirano H Yoshida H Suzuki T. Ratio and associated factors of dry mouth among community-dwelling elderly Japanese women. Geriatr Gerontol Int. (2011) 11:83–9. 10.1111/j.1447-0594.2010.00647.x 20807242

[15] Thie NM Kato T Bader G Montplaisir JY Lavigne GJ. The significance of saliva during sleep and the relevance of oromotor movements. Sleep Med Rev. (2002) 6:213–27. 10.1053/smrv.2001.0183 12531122

[16] Zhao H Ran S Huo W Gan K Li W. Xerostomia correlates with pain sensitivity in burning mouth syndrome patients. Sci Rep. (2025) 15:13191. 10.1038/s41598-025-97048-6 40240798 PMC12003751

[17] da Silva LA Teixeira MJ de Siqueira JT de Siqueira SR. Xerostomia and salivary flow in patients with orofacial pain compared with controls. Arch Oral Biol. (2011) 56:1142–7. 10.1016/j.archoralbio.2011.04.001 21612767

[18] Canfora F Calabria E Spagnuolo G Coppola N Armogida NG Mazzaccara C Salivary complaints in burning mouth syndrome: a cross sectional study on 500 patients. J Clin Med. (2023) 12:5561. 10.3390/jcm12175561 37685630 PMC10488611

